# Developing guidelines in low-income and middle-income countries: lessons from Kenya

**DOI:** 10.1136/archdischild-2017-312629

**Published:** 2017-06-05

**Authors:** Mike English, Grace Irimu, Rachel Nyamai, Fred Were, Paul Garner, Newton Opiyo

**Affiliations:** 1 Health Serviecs Unit, KEMRI-Wellcome Trust Research Programme, Nairobi, Kenya; 2 Nuffield Department of Medicine, University of Oxford, Oxford, UK; 3 Department of Paediatrics and Child Health, University of Nairobi, Nairobi, Kenya; 4 Maternal, Newborn, Adolescent and Child Health Unit, Ministry of Health, Nairobi, Kenya; 5 Kenya Paediatric Association, Nairobi, Kenya; 6 Centre for Evidence Synthesis for Global Health, Liverpool School of Tropical Medicine, Liverpool, UK; 7 Health Services Unit, KEMRI-Wellcome Trust Research Programme, Nairobi, Kenya

**Keywords:** Evidence Based Medicine, Paediatric Practice, Tropical Paediatrics, Guidelines

## Abstract

There are few examples of sustained nationally organised, evidence-informed clinical guidelines development processes in Sub-Saharan Africa. We describe the evolution of efforts from 2005 to 2015 to support evidence-informed decision making to guide admission hospital care practices in Kenya. The approach to conduct reviews, present evidence, and structure and promote transparency of consensus-based procedures for making recommendations improved over four distinct rounds of policy making. Efforts to engage important voices extended from government and academia initially to include multiple professional associations, regulators and practitioners. More than 100 people have been engaged in the decision-making process; an increasing number outside the research team has contributed to the conduct of systematic reviews, and 31 clinical policy recommendations has been developed. Recommendations were incorporated into clinical guideline booklets that have been widely disseminated with a popular knowledge and skills training course. Both helped translate evidence into practice. We contend that these efforts have helped improve the use of evidence to inform policy. The systematic reviews, Grading of Recommendations, Assessment, Development and Evaluation (GRADE) approaches and evidence to decision-making process are well understood by clinicians, and the process has helped create a broad community engaged in evidence translation together with a social or professional norm to use evidence in paediatric care in Kenya. Specific sustained efforts should be made to support capacity and evidence-based decision making in other African settings and clinical disciplines.

## Background

Evaluations of district hospital (first referral) level care for children and newborns conducted by WHO in multiple countries^1^ and at the national level in Kenya^2^ well over a decade ago highlighted that poor quality of care was common. Resources were inadequate and no clinical guidelines were available, although WHO had produced a text in 2000 to guide management of serious infections and malnutrition in hospitals.^3^ In addition, the degree to which such guidance was rigorously developed was questionable,^4^ and national clinicians may not adopt something perceived as developed by outsiders. Within this context in 2005 efforts began to create clear national clinical policies (referred to hereafter as guidelines) specifically targeting clinicians who provide admission care in district hospitals. This targeting recognised that availability of specifically trained paediatricians was very low and that more than 50% of hospital deaths occurred within 24–48 hours of arrival.^5^

**Figure 1:**
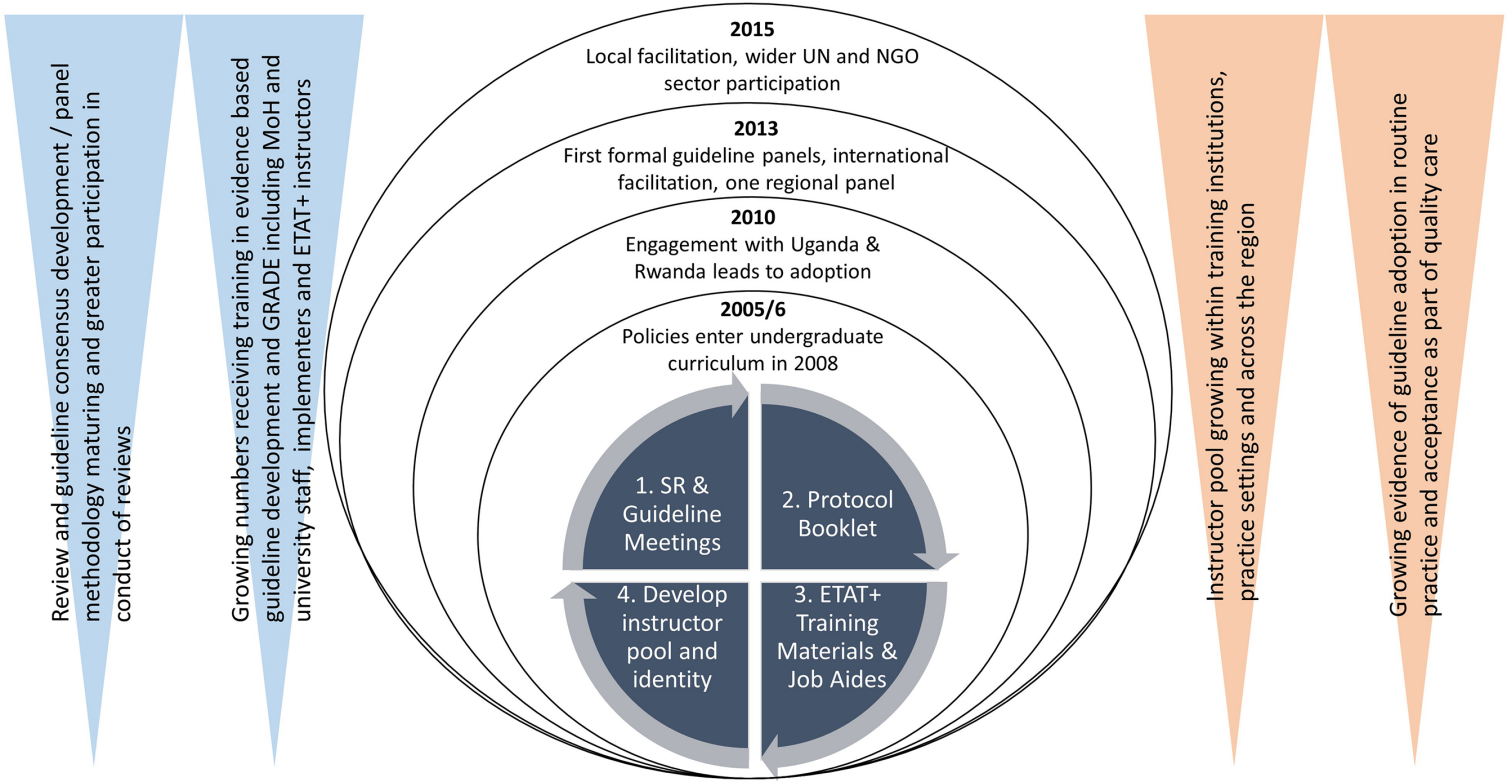
A diagrammatic representation of the evolution of the evidence-informed policy making progress. Each of four rounds of policy making, represented as rings for the years 2005, 2010, 2013 and 2015, included the conduct of systematic reviews (SR) linked to guideline meetings where multiple stakeholders were engaged in a consensus-building process to make policy recommendations based on the evidence. After the meetings, recommendations were formatted as protocols (algorithms) and included in a booklet to help disseminate policies. The policies also informed development and updating of a training course (ETAT+) that in turn helped create an instructor pool and a professional to identify evidence-informed practice. Over the period of 2005–2015, the technical procedures and level of engagement matured (blue-shaded triangles) while the number of policy champions and evidence of adoption also grew (orange-shaded triangles). MoH, Ministry of Health; NGO, non-governmental organisation; UN, United Nations, GRADE, Grading of Recommendations, Assessment, Development and Evaluation.

In table 1 and figure 1 we outline in a temporal sequence the evolution of the Kenyan guidelines’ procedural and technical developments and wider outputs. In table 2 we provide examples of the clinical guideline issues tackled. Specific features of the evolution are discussed linked to these periods with our own reflections offered on the progress made, potential lessons and implications for other countries.

**Table 1:** A summary of the evolution of the strategy for developing evidence-based clinical guidelines from 2005 to 2015

Year of activity	Topic selection and number of topics	Stakeholders involved	Technical preparation and materials developed	Format and duration of discussions	Guideline panellists	Decision-making process and documentation	Outputs	Policy changes and Outcomes
2005	14 topics selected based on morbidity and mortality of common inpatient conditions and identified areas of poor quality care in Kenya	Researchers completed reviews and set up meeting; clinical community engaged in the first ‘Child Health Evidence Week’.	Rapid, contextualised systematic reviews prepared, and presented at the meeting; reviews not provided to panel in advance	A local expert selected by researchers was given the systematic review and slides before the meeting and asked to present the evidence; presentation of evidence and discussion lasted about 1.5 hours per topic.	35 participants: mostly university paediatricians, some Medical Training College faculty (who trained non-physician clinicians), local researchers and MoH personnel	No formal consensus process; chair highlighted the evidence, existing WHO recommendation and potential adaptations, and facilitated agreement (assent); recommendation documented, but not the discussion	Four systematic reviews were published in peer-reviewed journals.	First national paediatric and neonatal guidelines for hospital care spanning major conditions; previously only available for malaria and HIV; although approved by the MoH in 2006, they were produced in small numbers until 2008 when 10 000 distributed
2010	2005 edition update: 11 topics focused on important new research in existing topics covered, and by requests of those teaching use of the guidelines	The researchers completed reviews but engaged with the MoH, the KPA and the UoN in coordinating the reviews and meeting	Rapid reviews produced with MoH or UoN staff jointly after GRADE training; reviews and SoF provided at the start of the meeting; participants were sent reviews for 3 key topics 4 weeks prior to the meeting as part of a trial^14^	Panel induction on GRADE (2 hours); evidence presentation and discussion 1.5 hours followed by a 2-hour facilitated discussion on recommendations; voting using a modified GRADE grid helped generate recommendations	60 participants: mostly paediatricians from two major medical schools, Medical Training College faculty, pharmacists with procurement roles, MoH personnel and local WHO officers	Non-voting facilitator guided discussions, the group examined the evidence before draft recommendations were discussed, taking account of context, feasibility, and preferences; blinded vote on draft recommendations with results reviewed 1 day later in 30 min to make final recommendations	Updated 2005 Guidelines; five reviews published in peer-reviewed journals and all reviews made available on a website; formal evaluation of the process published^14 15^	11 policies revised; 12 000 copies of updated guidelines disseminated with the MoH; in-service, undergraduate and postgraduate training were updated to reflect changes
2013	Three topics selected: two where major trials published (intravenous fluids in shock, cord care), and one for a drug entering practice without evaluation of benefits/harms (hydroxyurea in sickle cell disease)	The research team, the MoH and the KPA organised for three specific reviews to inform three topic-specific guideline panels each to convene at the annual national paediatric conference	Researchers and seconded members of the MoH and UoN completed reviews with SoF tables; support from the Cochrane Infectious Diseases group; reviews were sent 4 weeks in advance of the panel meeting with electronic copies of original references	Panel induction on GRADE (2 hours) before each panel started work; panels chaired by one non-expert member; evidence presented by review author and discussed, panel decided level of certainty of effects (1.5–2 hours), followed by discussions informed by the DECIDE framework on risks and benefits, feasibility and acceptability of possible recommendations (4–8 hours); facilitation by a senior researcher and technical experts not involved in final decisions	Specific panels selected for each topic (3 in 2013 and 4 in 2015) each with 16–20 panellists; core personnel from the MoH, representatives of regulatory and training bodies for clinical officers and nurses, and the national medicines procurement body (also representing pharmacists) sat on all three panels; they were joined by 8–10 additional panel-specific members drawn from among topic-specific experts, and medical and nursing practitioners from typical district hospitals	Meetings were preceded by disclosure of interests; chairperson and facilitator sought to foster participation, full exchange of views and then develop consensus; the participation, process, key discussion points and recommendations were documented in a guideline panel report that named all participants and reported their roles	Reports and draft recommendations made publicly available on a website and given to the MoH to make final decision on recommendations; revised national guidelines produced after MoH finalised recommendations 2013: Evidence, discussions and draft recommendations immediately disseminated at conference 2013: Three reviews published in peer reviewed journals 2015: MoH held multistakeholder donor meeting to promote and get support for new pneumonia policy	Revised guidelines including three new recommendations were published and 12 000 copies distributed before the end of 2013; neighbouring countries used evidence materials and amended guidelines
2015	Pneumonia trial initiated given insufficient evidence in 2010; on completion a guideline panel was constituted; two neonatal care topics not reviewed since 2005, one topic concerned guidance on a technology (CPAP) being introduced in an ad hoc fashion by hospitals	As in 2013 the research team worked with the MoH, the KPA and the UoN; 6 paediatricians joined researchers to form systematic review teams 6 months in advance of the planned guideline meetings	Four systematic reviews spanning guideline-related questions; reviews done by emerging junior team supported by an experienced Kenyan researcher and one by international collaborators; all reviews had GRADE SoF tables; these were sent out to panellists 4 weeks before panel meetings					Revised protocols including four new recommendations were published and 12 000 copies distributed in 2016

CPAP, continuous positive airway pressure; MoH, Ministry of Health, Kenya Paediatric Association (KPA), University of Nairobi (UoN), Grading of Recommendations, Assessment, Development and Evaluation (GRADE), Summary of Findings (SoF)

**Table 2:** Examples of evidence-informed clinical guideline areas covered during 2005–2015

Disease-specific treatments	Supportive care	Treatment of common emergencies	Prevention
Severe malaria: Quinine loading doses (2005), artesunate as first-line therapy (2010) Pneumonia: First-line antibiotic treatment (2005); Amoxil for indrawing pneumonia (2010 and 2015) Meningitis: First-line antibiotic treatment (2005); steroids (2010) Diarrhoea: Zinc treatment (2010) Neonatal sepsis: Once daily gentamicin (2005); sickle cell disease: hydroxyurea prophylaxis in under 5s (2013)	Feeding: Regimens for F75/F100 in severe malnutrition (2005); use of RUTF in malnutrition (2010); time of initiation of feeding in preterm babies (2005 and 2015); use of breast milk fortifiers (2005 and 2015); rate of increasing feeds (2010 and 2015) Respiratory support: Treatment of neonatal apnoea with caffeine (2010); CPAP in neonatal respiratory distress (2015)	First-line anticonvulsant regimens in children (2005) and newborns (2010); bolus glucose for possible hypoglycaemia (2005); fluid bolus in severely ill children (2013); newborn resuscitation (2005 and updated in line with new international guidance in 2010)	Alcohol handrubs for infection control (2010); chlorhexidine cleaning of the umbilical cord (2013); antibiotic prophylaxis to prevent neonatal sepsis in at-risk babies (2015)

CPAP, continuous positive airway pressure, RUTF, ready to use therapeutic foods.

### The four guideline meetings 2005–2015

In 2005 the Cochrane Collaboration and the UK’s National Institute for Health and Care Excellence had been established for only 13 and 6 years, respectively, and the WHO had no specific procedures to incorporate research into the guideline development process table 1.^4^ Recognising the need for improved access to usable guidance,^2^ a small research team in Kenya was formed, endorsed by the Ministry of Health, with two main aims. First was to improve the use of research evidence in creating national clinical policies. This was manifest in 2005 as an ambitious aim to conduct systematic, contextualised evidence summaries for 14 clinical topics. The second was to engage government personnel and those with a major potential role in promoting ownership and adoption of policy in a shared decision-making process. The team initially trained itself in review methodology before engaging with an emerging, voluntary international grouping^6^ that subsequently went on to support the production of the WHO *Pocketbook of Hospital Care for Children*,^7^ to which the Kenyan group made contributions.

Initial reviews (eg, refs 8 9) were used to inform discussions at a first ‘Child Health Evidence Week’ held in June 2005. At this meeting 35 participants (table 1) spent an entire week briefly exploring and then debating the evidence before agreeing policy recommendations on the treatment of pneumonia, malaria, diarrhoea, meningitis, malnutrition, neonatal sepsis, feeding of the preterm and common complications of illness (see table 2). The participants also approved in draft format treatment algorithms that would become a 36-page booklet of guidelines, a simple formulary and charts to support paediatric prescribing of medicines, fluids and feeds. Importantly, the focus was to provide guidance not for the professional paediatric community but as a basic standard for acute care provided at the referral level by non-specialist clinicians. This booklet, published by government, was also a key tool used in a popular training course (ETAT+) that aimed to improve skills and promote guideline adoption.^10^ ETAT+ training was first used in 2006 as part of a multifaceted intervention strategy shown to improve district hospital care.^11^ It also became integrated into undergraduate and postgraduate paediatric training from 2008^12^ in Kenya’s largest medical school helping to popularise the guidelines.

With increasing use of the guidelines and based on feedback from an emerging group of ETAT+ instructors, demand for their revision grew. The first revision was undertaken in 2010 (table 1). This incorporated for the first time in Kenya training in and use of the GRADE approach^13^ to inform evidence synthesis and culminated in a second Child Health Evidence Week. In 2010 non-research paediatricians were engaged in reviews and given basic training in review methodology and GRADE. At the guidelines meeting there was a greater focus on structured, facilitated discussion to contextualise evidence and develop draft recommendations that were voted on using a modified GRADE grid and individual, anonymous voting slips. Voting patterns were presented back to all participants used to create draft recommendations submitted to the Ministry of Health. Visitors from Uganda, Rwanda and Tanzania observed the meeting that was facilitated by a senior researcher. Aspects of the entire process were evaluated^14 15^ with the assistance of international experts. The output was a revised set of guidelines subsequently published and disseminated (table 1).

Efforts to revise guidelines in 2013 were prompted by publication in late 2011 of a large East African trial, with results that contradicted prevailing thinking on fluid management of very sick children.^16^ With little guidance forthcoming from WHO, guideline champions and ETAT+ instructors (now an important practice grouping)^17^ were frequently questioned on what was the best course of action. A local systematic review^18^ and a guideline meeting to include participants from Uganda and Rwanda, countries where Kenyan guidance was also informing practice, were planned. At the same time important questions were being raised by the paediatric community about the use of chlorhexidine to clean the umbilical cord and the risks and benefits of the emerging use of hydroxyurea to treat sickle cell disease in young children. Therefore, three systematic reviews were undertaken with prominent roles of personnel from the Ministry of Health and University of Nairobi supported by the research team^18–20^ and the Cochrane Infectious Diseases group.

### Development of the approach

The process of translating this evidence into policy changed considerably in 2013 (table 1). Three topic-specific and more formally conducted guideline panels, each with 16–20 selected panellists, were convened at the annual national paediatric conference (table 1). Each panel met for a whole day after receiving the systematic review and the primary literature 4 weeks in advance of their meeting. For the first time panels formally documented disclosure of interests and their judgement about the level of certainty of effects, and used the DECIDE framework to explore the risks and benefits, feasibility and acceptability of possible recommendations^21^ until a consensus was reached. The participation, process, key discussion points and recommendations were documented, made publicly available online and were passed to the Ministry of Health. Immediate dissemination of key findings and draft recommendations occurred at the national conference, and policies were changed and guideline booklets disseminated before the end of 2013. (WHO recommendations on the subject of fluid management were not published until 2016).^22^

The model for guideline development in 2013 was used again in 2015. For childhood pneumonia uncertainty in the global evidence examined in 2010^23^ had prompted conduct of a local pragmatic trial.^24^ With these new results a panel was constituted and developed draft recommendations for a substantial change in treatment policy before the trial results were published. In recognition of poor quality of care^25^ and persistently high neonatal mortality,^26^ three neonatal guideline panels were also constituted (for topics see table 1). Once again emerging use of new technology, continuous positive airway pressure for newborns, which was entering district hospital practice without review of benefits and harms, prompted development of local guidance.

### Reflections

Initial efforts in 2005 at review of evidence and its translation to policy through discussion and building consensus (informally) had limitations by the standards emerging at the time.^27^ However, even in 2005 a rigorous and systematic approach to identifying literature was employed, with search strategies based on the PICO framework (population, intervention, control and outcome), although searches have largely been restricted to the English-language literature. Importantly evidence syntheses included observational studies where necessary given the lack of randomised trial evidence for many questions of relevance to low-income countries (LICs). The process was further enhanced from 2010 by employing specific software (GRADEpro) that documents and makes transparent the appraisal decisions made for each included article, information that underpins the final determination of certainty in evidence of effects. The guidelines created have influenced undergraduate and specialist training in Kenya (and elsewhere) from 2008 to date and resulted in a high proportion of graduating doctors in Kenya carrying knowledge of the guidelines to their workplace complementing other modes of dissemination.^12^ The emergent broad approach linking systematic review, engagement in evidence appraisal, consensus-based decision making, development of guideline booklets and an adult education-oriented interactive form of knowledge and skills training represent an overarching effort to translate evidence to policy and subsequently into practice, with evidence of some success.^17^ Trainers on skills courses have become in many ways a community of practice receptive to evidence-based policy change.^12^ The presence of observers from neighbouring countries in 2010 and again in 2013 helped support wider use of these evidence-informed policies and linked training in Uganda, Rwanda and other countries,^12 28^ extending this community and increasing impact.

Technical and procedural improvements were made over time (table 1). These included methodological developments in conduct of systematic reviews, GRADE training, declaration of interests, debate and agreement on the certainty in evidence of effects from a health system perspective, facilitation of discussion informed by the DECIDE framework to develop consensus-based recommendations, and use of formally constructed panels representing multiple constituencies. As this process evolved, so did engagement with and participation of local academic clinicians, the national paediatric association, health professions’ regulatory bodies, and importantly the Ministry of Health spanning multiple departments and specific secondment of personnel (table 1 and figure 1). An important element of the process, which was new to most involved, was a formal process of decision makers declaring any potential interests in the outcome of the guideline discussions. As many LIC academics rely on sponsorship to attend international conferences and as the private sector is becoming more prominent as a research funder in LIC, such disclosures are important. These together with a publicly available listing of panel members and a summary of their panel discussions are a critical element of good practice in guideline development supporting the transparency and credibility of final recommendations.

In 2010 specific efforts were made to evaluate preferences for or effects of different forms of presenting evidence from systematic reviews.^14 15^ These evaluations indicated very limited familiarity with evidence synthesis or critical appraisal of research literature among panellists prior to meetings. They also indicated a preference for contextualised narrative presentations of synthesised evidence and that limited time was spent reading reviews in preparation for panel meetings, emphasising the value of increasingly structured presentation and discussion of the available evidence.^14^ Despite these apparent limitations qualitative work suggested active and pertinent engagement with the evidence during discussions, diversity of opinions and an ability to accept a consensus. They also suggested an enthusiasm for participation, greater appreciation of the value and limitations of evidence, and an emerging sense of ownership of the process,^15^ perhaps linked to a sense that roles were not just to endorse global recommendations.^23^ In 2012, 60% of clinicians encountered in a survey of 22 hospitals had copies of the guidelines^29^ and there was evidence of substantial practice change.^17^

Limitations remain however. Currently mechanisms for patient engagement in the policy process and data on costs and formal cost-effectiveness analyses are largely absent in many LICs. We suspect, as in Kenya, that there is often no structured process for identifying local priority topics in LIC. Often (and appropriately) global issues may drive the process or as in Kenya it may be somewhat reactive, drawing on informal communication from policy makers, the agenda of technical partners or researchers. Despite these limitations the model that has evolved in Kenya uses internationally defined good practice, to the extent that is feasible, and is probably one of the most structured and participatory approaches to national guideline development in a LIC in Sub-Saharan Africa. The process has provided the platform for a complete cycle of decision making spanning a request for better evidence,^23^ conduct of a pragmatic trial^24^ and a new round of decision making. It has also helped foster wider awareness of how evidence can inform policy and the technical aspects of this process.^15^

It is not enough to make even well-informed decisions. These must be linked to a clear plan for disseminating guidance and ideally ensuring such new guidance can rapidly reach those providing and receiving health worker training.

Possible lessons for other LIC embarking on this journey are summarised in box 1. More generally we compare the evolution of our efforts with what is known about the science of using evidence to inform policy that proposes six major mechanisms that influence success.^30^ The approach we describe spans five, and our experience would suggest that countries consider all of these from the inception of their own efforts. These are building awareness of and a positive attitude towards evidence-informed decision making, providing communication of and access to the evidence, providing interaction between decision makers and researchers, supporting decision makers in developing the skills to make sense of the evidence, and influencing the structure and process of decision making.^30^ We also suggest that sustained effort to build a partnership spanning research, government, academia, professional associations and practitioners is important. Over a decade more than 120 people were engaged in the decision-making process and more than 25 contributed to the conduct of systematic reviews. Linkage with a training course and its expert trainers, some involved in guideline panels, has helped extend the community involved in the evidence translation process. Broadly we suggest this overarching approach has helped evidence translation by creating a social or professional norm among both decision makers and practitioners to use evidence, has been an effective strategy for awareness raising at scale and has helped reshape professional identity towards acceptance of common practice standards.^30^

Box 1 **Initiating a process of national guideline development—drawing lessons from the Kenyan experience**

- A credible guideline process will probably require at least two or three individuals who are familiar with the technical process of systematic review and use of the GRADE approach. This small team can help present and explain the evidence to a guideline panel so it is formally and critically discussed (a process that in itself builds ownership). Ideally one person should have more specific training in clinical epidemiology. Joining a team undertaking a formal Cochrane review, taking short courses in review methodology, and building links with the growing international community involved in evidence synthesis and evidence-informed decision making can provide a useful training for this small team while helping link them to a wider community of practice.
- Beginning with a set of specific target conditions and an identified guidelines user group may help focus efforts. We tackled care for common, acute conditions aiming to define first-line treatment strategies. Doing this we were able to build on existing international efforts to improve ‘case management’. By targeting most junior providers, we were not attempting to replace expert opinion which may have resulted in resistance to guidance, but to standardise practice in those with little training. An initial country effort might only tackle one or two pressing guideline questions as a demonstration of the process and its value.
- Identifying topics for guideline development where concerns already exist about routine practice and outcomes, where there is uncertainty about the value of a new technology or treatment, or where new research findings raise questions about existing practice may help create demand for consensus in the professional community and provide opportunities for initiating a guideline development process.
- Engaging appropriate stakeholders is clearly important from the start. Those involved in the decision making should have the authority and credibility to support any guideline’s claims to be a national (or regional) recommendation. Government, professional associations, academic institutions and potentially regulatory bodies may thus need to be involved. At the same time it is important to engage those involved in frontline practice. A guideline panel of 16–20 seems adequate to span all these groups. They must be given a real opportunity to discuss the evidence and reach consensus. By providing well-written reading materials and with skilled facilitation, 1 day seems sufficient to discuss a single topic in detail even when panellists themselves have little experience in evidence appraisal and use.

In conclusion, our report contributes to a relatively small literature exploring the process of national guideline development in LIC.^31–33^ Over time a trade-off between greater attention to complying with international recommendations for translating evidence into policy that increases costs and limits the number of topics that can be tackled^34^ has become apparent. Clearly rigour and transparency are important. However, efficiencies can be gained by countries sharing systematic reviews so they do not need to be repeated, a clear aim of organisations such as the Cochrane Collaboration and WHO. This does not obviate the clear requirement for making country-relevant decisions locally to take into account context and national values and preferences and promote ownership. While global guidance provides a useful benchmark, this should inform but not replace such processes. Our experience suggests that long-term partnership may enable successful, timely and credible national guideline development. Sustaining such approaches and embedding them within local institutions should be considered a part of the health system strengthening agenda.
